# Prevalence and associated factors of social phobia among high school adolescents in Northwest Ethiopia, 2021

**DOI:** 10.3389/fpsyt.2022.949124

**Published:** 2022-10-25

**Authors:** Girum Nakie, Mamaru Melkam, Getachew Tesfaw Desalegn, Tadele Amare Zeleke

**Affiliations:** Department of Psychiatry, College of Medicine and Health Science, University of Gondar, Gondar, Ethiopia

**Keywords:** prevalence, social phobia, adolescents, Ethiopia, associated factors

## Abstract

**Background:**

Social phobia is the third most common mental illness in the world. It harms educational achievement by increasing school absentees and prevents students to participate in class, and this leads to a significant impairment of the emotional, psychological, social, and physical wellbeing of students. The research done regarding social phobia and associated factors among high school students in low- and middle-income countries is limited. Therefore, this study aims to assess the prevalence and associated factors of social phobia among adolescents and have a pivotal role in further investigation.

**Objectives:**

To assess the prevalence and associated factors of social phobia among high school adolescents in Northwest Ethiopia, 2021.

**Materials and methods:**

An institutional-based cross-sectional study was conducted from 15 April to 14 May 2021, by using a simple random sampling technique to select a sample of 936 participants after proportional allocation to the six high schools. Social phobia was assessed by using the social phobia inventory (SPIN), independent variables like social support were assessed by Oslo social support scale, substance-related factors by ASSIST, and the rest of the other factors were assessed by structured questionnaires. Binary and multivariate analyses were done to identify factors associated with social phobia. Statistical significance was declared at a 95% confidence interval (CI) of *p*-value less than or equal to 0.05.

**Result:**

The prevalence of social phobia among adolescents was found to be 40.2% (95% CI 37.0 to 43.4%). In the multivariable analysis, female sex (AOR = 1.374, 95% CI = 1.016, 1.858), poor social support (AOR = 2.408, 95% CI = 1.660, 3.493), having known chronic medical illness (AOR = 2.131, 95% CI = 1.173, 3.870), having a history of mental illness in the family (AOR = 1.723, 95% CI = 1.071, 2.773), and is highly risky alcohol user (AOR = 1.992 95% CI 1.034, 3.838) were factors significantly associated with social phobia symptoms.

**Conclusion:**

The overall prevalence of SP among adolescents was high. Therefore, early detection and adequate intervention are crucial to reducing the overall burden of social phobia among adolescents.

## Introduction

According to DSM-V, social phobia (also referred to as social anxiety disorder) is defined as intense, persistent fear, or anxiety of social situations in which the individual may be scrutinized by others and this situation interferes significantly with routines, academic functioning, and social activities (1).

In turn, social phobia in a school is the response pattern of the high level of arousal, avoidance, and escape behavior that is elicited by stressful school environment like speaking to the class, being rejected by peers, and answering questions of the teacher that the student perceives as negatively evaluated (1, 2).

Social phobia is the third most common mental illness in the general population, and even the most illness of adult social phobia onset was during adolescence (3, 4). Research conducted in seven countries showed that the lifetime prevalence of social phobia varies across the world from a range of 22.9–57.6% (5). In an international community survey across different 13 countries, the magnitude of SP was 4% and it was highly prevalent among females and young age groups (6). In a different study conducted in Africa, the prevalence of social phobia ranges from 10.3 to 76.4% (7–9), and in Ethiopia was 27.5% (10).

Different factors affect SP. These include low educational status, substance use, poor daily functioning, and unstable life, (6, 11–13) which lead to a remarkable impairment of emotional, psychological, and social wellbeing (7–9, 14). The risk of SP also among high school students is higher than among those who have poor academic performance, alcoholic drinkers, female gender, being living in rural, have young ages, victimization, comorbid chronic medical illness, and have a past and family history of mental illness (10, 15–20).

Therefore, students with SP tend to have faced different problems. Such impaired social interactions like a risk to have fewer friends, feeling lonely, disappointed over missed opportunities for friendship, and hiding from others, might prevent students from discussing with friends in the classroom, going to a party, and joining different enjoying activities, and these problems extend through adulthood (13, 21).

In a study conducted in Sweden and Poland, SP negatively affects students’ educational performance, might keep a person from volunteering to answer in class, reading aloud, giving a presentation, and avoiding oral questions, and all of this leads to poor academic performance (22, 23). Students with social phobia were also consistently more likely to experience a variety of psychological problems, concurrent medical, and mental illness always occurs following social phobia, and this comorbidity may lead to poor prognosis and tends to impaired family relationships and commit suicide (13, 17, 24). A cross-sectional study conducted in developing countries including Ethiopia suggests that the risk for substance abuse like misuse of alcohol and other substance dependence is high in socially anxious students (24–26).

While SP among high school students has been relatively researched in developed countries, very few studies are available in developing countries including Ethiopia. In Africa, though SP was researched among university students, according to our research engine, there was only one published research in Ethiopia, but not included grade nine and ten students. Therefore, this study was conducted to assess the prevalence of SP and various factors that might be led to early interventions for further obstacles among high school adolescents. For instance, this study will give important recommendations to reduce the risk factors of social phobia and may contribute students to improving the status of their academic performance. Furthermore, the result of this study will provide information for health professionals to design appropriate solutions for the problem.

## Materials and methods

An institutional-based cross-sectional study design was conducted from 15 April to 14 May 2021, among high school adolescents in Northwest Ethiopia. The study was conducted among six areas of high schools in Northwest Ethiopia and covered 1,018.11 km^2^. There were six governmental high schools that offered a total of 12,977 students, of these 6,587 are males and the rest are females, one primary hospital, and six health centers in the district, but no private schools. All high school adolescents who have been learning in Northwest Ethiopia were the source population. Thus, all high school students who were presented in class during data collection time were study populations. All high school students who attended a class during data collection time were included in the study, whereas students who were unable to communicate due to acute illness during data collection time at schools were excluded.

## Sample size determination and procedure

The sample size was determined by assuming single population formula with the assumptions: The prevalence of social phobia was 27.5% (10), 95% confidence interval (CI), margin error of 3%, and 10% non-response rate. Accordingly, the final sample size of 936 students was used.

High school students in the area of the study were stratified based on their grades as grade nine, grade ten, grade eleven, and grade twelve. As data obtained from the Education office indicated that the total number of high school students during data collection was 12,977 (grade nine = 3,598, grade ten = 4,037, grade eleven = 2,940, and grade twelve = 2,402). Then, proportional allocation of study subjects for each stratum (grades) was calculated, and 260, 291, 212, and 173 high school students were drawn from grade nine, grade ten, grade eleven, and grade twelve, respectively. Finally, a computer-generated lottery method was used to select study participants from each given strata.

### Operational definitions

Social phobia: From the social phobia inventory (SPIN) tool assessment, students who scored 20 and above considered to be social phobia (10).

Social support: From Oslo three-item scales, students who scored 3–8 on poor social support, 9–11 on moderate social support, and 12–14 on strong social support (27).

Current substance use: Measured by the ASSIST scale for the past 3 months. Therefore for alcohol, students who scored 0–10 were low risky, 11–26 moderate users, and 27 and more highly risky drinkers, whereas for current khat and cigarette use participants scored 0–3 low risky, 4–26 moderate risky, and 27 and more highly risky users (28).

Performance (average academic score): First-semester average results of students who scored 49% and below are considered to be poor, 50–74% sufficient, 75–84% good, and 85% and above very good academic performances (29).

Age: From WHO age classification, declared that adolescents aged 10–19 years and adults aged 20 years old and above (30).

### Data collection tools

Data were collected using a structured self-administered questionnaire that has five parts: In part one, socio-demographic characteristics such as age, sex, grade, and the like were collected by using structured socio-demographic questionnaires. In the second part, an outcome variable prevalence of social phobia was assessed by using the social phobia inventory (SPIN). The sensitivity and specificity of the SPIN were 82.2 and 77.6%, respectively, as it was validated in Nigeria. The positive and negative predictive values were 80% each (31). It was used among colleges and high school students in different countries including Ethiopia (10, 14, 20, 31). Part three clinical factors like family history of mental illness and history of other mental illness, suicide ideation and attempt, and chronic medical illness were assessed by structured yes/no questions. The fourth part substance-related factors, which comprise substance use for its assessment of which is currently used and ever used, were adapted from the ASSIST (Alcohol, Smoking, and Substance Involvement Screening Test). It is a well-validated instrument developed by the World Health Organization (28, 32). Finally, in part five, psychosocial factors were assessed by both standardized tools and structured questionnaires: Therefore for social support, the OSLO three-item social support scale was used (27), whereas for both social media and mass media usage was assessed by structured yes/no questions.

To control the quality of data, the questionnaire was initially prepared in English, then translated into the Amharic language, and finally back to in English by two language experts and psychiatrists appropriately. The training was given to data collectors and supervisors, and each completed questionnaire was checked and the necessary feedback was also offered to interviewers the following morning. The questionnaire was pretested 1 week before the actual data collection time on 5% (*n* = 47) of the study who were not included in the main survey. Therefore, the dependent variable tool assessment (SPIN) Cronbach alpha was 0.899. Based on the feedback obtained from the pre-test, an appropriate modification was made to the questionnaire.

The collected data were coded, edited, entered, and checked into the computer using EPI data version 4.6.02 and imported to SPSS version 25 to generate descriptive statistics: means, standard deviation, frequency, and percentages. To determine an association between dependent and independent variables, adjusted odds ratios were used using logistic regression and the significance level was determined using a confidence interval of 95%. Bivariate and multivariate logistic regression was used to identify the independent predictors of social phobia. Each independent variable was separately entered in the bivariate analysis. The variables with a *p*-value of less than 0.25 on bivariate analysis were entered into multivariate analysis. The variables that showed statistically significant association with a *p*-value of less or equal to 0.05 on logistic regression were considered to be predictors of social phobia.

## Results

### Socio-demographic characteristics of participants

Data were obtained from 876 high school adolescents with a response rate of 93.6%. The mean age of the participants was 18.49 ± 1.706, ranging from 15 to 25 years old, and 618 (70.5%) of them were within 15–19 years old. More than half (55.4%) and 709 (80.9%) of the students were females and originally from rural areas, respectively. The majority of the students were single 815 (93.0%) and living with their two parents 682 (77.9%) (Table 1).

**TABLE 1:** Socio-demographic characteristics of participants among high school adolescents in Northwest Ethiopia (*n* = 876), 2021 Gorgonian Calendar (GC).

Variables	Categories	Frequency	Percent
Sex	Male	391	44.6
	Female	485	55.4
Age	≤19	618	70.5
	≥20	258	29.5
Place of upbringing	Rural	709	80.9
	Town	167	19.1
Living arrangements	Both parents (father and mother)	682	77.9
	Single parents (father or mother)	86	9.8
	Alone	55	6.3
	Relatives	53	6.1
Father educational status	Unable to read and write	239	27.3
	Can read and write but not attending formal education	490	55.9
	Learned 1–8 grade	80	9.1
	Secondary education	34	3.9
	Diploma and above	33	3.8
Mother educational status	Unable to read and write	620	70.8
	Can read and write but not attending formal education	185	2.1
	Learned 1–8 grade	39	4.5
	Secondary education	17	1.9
	Diploma and above	15	1.7
Marital status	Married	50	5.7
	Single	815	93.0
	Widowed	11	1.3
Grade	9	241	27.5
	10	268	30.6
	11	205	23.4
	12	162	18.5
Academic performance	≤49.99	62	7.1
	50–74.99	165	18.8
	75–84.99	626	71.5
	≥85	23	2.6
Absenteeism	Yes	92	10.5
	No	784	89.5

### Clinical characteristics of the respondents

Out of the total participants, 63 (7.2%) have a history of mental illness, 90 (10.3%) have lifetime suicidal ideation, and 55 (6.3%) students had known chronic medical illnesses. Therefore from 55 chronic medical illnesses, the highest was epilepsy (32), the second was hypertension (8), and the list observed was asthmatics (2), and five students had cardiac problems, four students had HIV, and three students had diabetes mellitus (Table 2).

**TABLE 2:** Clinical characteristics of participants among high school adolescents in Northwest Ethiopia (*n* = 876), 2021 Gorgonian Calendar (GC).

Variables	Categories	Frequency	Present
Having known chronic medical illness	Yes	55	6.3
	No	821	93.7
Having known mental illness	Yes	63	7.2
	No	813	92.8
Lifetime suicidal ideation	Yes	90	10.3
	No	786	89.7
Twelve-month suicidal ideation	Yes	51	5.8
	No	825	94.2
Lifetime suicidal attempt	Yes	42	4.8
	No	834	95.2
Twelve-month suicidal attempt	Yes	26	3.0
	No	850	97.0
Family history of mental illness	Yes	93	10.6
	No	783	89.4

#### Substance-related characteristics

Regarding substance use, out of the students, 404 (46.1%) were drinking alcohol at least once in their lifetime, whereas khat and cigarette lifetime users were 78 (8.9%) and 57 (6.5%), respectively. About four in five of the students (80.3%) were low risky alcoholic drinkers, whereas moderate and highly risky alcoholic drinkers were 129 (14.7%) and 44 (5.0%), respectively, (Table 3).

**TABLE 3:** Substance-related description for participants among high school adolescents in Northwest Ethiopia (*n* = 876), 2021 Gorgonian Calendar (GC).

Variables	Categories	Frequency	Percent
Alcohol	Low	703	80.3
	Moderate	129	14.7
	High risk	44	5.0
Khat	Low	845	96.5
	Moderate	19	2.2
	High risk	12	1.4
Cigarette	Low risk	853	97.4
	Moderate	17	1.9
	High risk	6	0.7

### Psychosocial characteristics of participants

Of the participants, about one-third of students have strong social support 293 (33.4%), whereas students who had moderate and poor social support were 339 (38.7%) and 244 (27.9%), respectively. Regarding media usage, 172 (19.6%) of the participants were using social media, and 633 (72.3%) were using mass media.

#### Prevalence and associated factors of social phobia

In this study, the overall prevalence of social phobia among high school adolescents was shown that 352 (40.2%) with a 95% CI of 37.0 to 43.4%.

Female sex, age less than or equal to nineteen, place of the upbringing of students, father’s educational status, marital status, grade, living arrangements, academic performance, absence from class, history of known chronic medical illness, history of mental illness, family history of mental illness, current alcohol drinking, current khat chewing, and social support were factors associated with SP at *p*-value less than 0.25 in binary logistic regression. Finally, multivariate analysis revealed that female sex, having a history of known chronic medical illness, family history of mental illness, highly risky current alcohol drinkers, and poor social support were found to be significantly associated with a social phobia with 95% of CI and at *p*-value less than or equal to 0.05.

Female adolescents were 1.4 times more likely to develop SP as compared with male adolescents (AOR = 1.374, 95% CI = 1.016–1.858), and adolescents who had a history of known chronic medical illness were about 2 (AOR = 2.131, 95% CI = 1.173–3.870) times to develop SP when compared with those who had no medical illness. Another associated factor with SP was having family history of mental illness (AOR = 1.723, 95% CI = 1.071–2.773) which is 1.7 times more odds to have SP than those who had not. Current alcohol drinking is also associated with social phobia (AOR = 1.992, 95% CI = 1.034–3.838). The odds of having social phobia were two times more prevalent in highly risky current alcohol drinkers as compared to low risky alcohol drinkers, and adolescents who had poor social support were about 2.4 times to develop SP when compared to those who had strong social support (AOR = 2.408, 95% CI = 1.660–3.493) (Table 4).

**TABLE 4:** Bivariate and multivariate analyses of factors associated with social phobia (SP) among high school adolescents in Northwest Ethiopia (*n* = 876), 2021 Gorgonian Calendar (GC).

Variables	Category	Social phobia		COR and 95% CI	AOR and 95% CI	*P*-value
		Yes	No			
Age	≤19	257	361	1.221 (0.906–1.648)	1.036 (0.733–1.465)	0.840
	≥20	95	163	1	1	1
Sex	Female	**211**	**274**	**1.365 (1.039–1.795)**	**1.374 (1.016–1.858)**	0.039*
	Male	141	250	1	1	1
Upbringing	Rural	295	414	1.375 (0.966–1.957)	1.221 (0.830–1.795)	0.310
	Town	57	110	1	1	1
Living arrangements	Relatives	24	29	1.302 (0.742–2.285)	1.286 (0.705–2.347)	0.413
	Alone	24	31	1.218 (0.700–2.121)	1.360 (0.758–2.441)	0.303
	Single parents	39	47	1.306 (0.831–2.051)	1.294 (0.802–2.088)	0.291
	Two parents	265	417	1	1	1
Father’s educational status	Unable to read and write	100	139	1.655 (0.754–3.630)	1.852 (0.771–4.451)	0.168
	Can read and write but not attend formal education	198	292	1.560 (0.726–3.348)	1.836 (0.784–4.304)	0.162
	Learned 1–8 grade	29	51	1.308 (0.547–3.125)	1.542 (0.592–4.018)	0.375
	Secondary education	15	19	1.816 (0.665–4.959)	1.953 (0.657–5.804)	0.228
	Diploma and above	10	23	1	1	1
Marital status	Divorced	6	5	2.550 (0.676–9.615)	1.813 (0.435–7.557)	0.414
	Single	330	485	1.446 (0.785–2.662	1.302 (0.679–2.494)	0.426
	Married	16	34	1	1	1
Grade	9	105	136	1.278 (0.851–1.921)	0.994 (0.632–1.564)	0.980
	10	112	156	1.189 (0.797–1.773)	1.084 (0.690–1.705)	0.726
	11	74	131	0.935 (0.610–1.433)	0.787 (0.494–1.251)	0.311
	12	61	101	1	1	1
Academic performance	Poor	13	10	1.924 (0.731–5.066)	1.584 (0.566–4.434)	0.381
	Sufficient	256	370	1.024 (0.602–1.743)	0.920 (0.518–1.636)	0.777
	Good	58	107	0.802 (0.440–1.461)	0.767 (0.403–1.459)	0.419
	Very good	25	37	1	1	1
Class absence	Yes	43	49	1.349 (0.84–2.082)	1.396 (0.876–2.224)	0.160
	No	309	475	1	1	1
History of chronic medical illness	Yes	**34**	**21**	**2.561 (1.460–4.491)**	**2.131 (1.173–3.870)**	0.013*
	No	318	503	1	1	1
History of mental illness	Yes	30	33	1.386 (0.829–2.318)	0.832 (0.468–1.479)	0.531
	No	322	491	1	1	1
Family history of mental illness	Yes	**50**	**43**	**1.852 (1.202–2.854)**	**1.723 (1.071–2.773)**	0.025*
	No	302	481	1	1	1
Alcohol	Higher risk	**25**	**19**	**2.097 (1.133–3.882)**	**1.992 (1.034–3.838)**	0.04*
	Moderate risk	56	73	1.223 (0.836–1.788)	1.145 (0.761–1.722)	0.517
	Low risk	271	432	1	1	1
Khat	High risk	7	5	2.121 (0.668–6.737)	1.937 (0.581–6.455)	0.282
	Moderate	9	10	1.363 (0.548–3.391)	1.692 (0.638–4.484)	0.290
	Low risk	336	509	1	1	1
Social support	Poor	**133**	**111**	**2.618 (1.840–3.725)**	**2.408 (1.660–3.493)**	0.001***
	Moderate	127	212	1.309 (0.940–1.821)	1.343 (0.949–1.901)	0.096
	Strong	92	201	1	1	1

***p-value < 0.001, **p-value (0.001–0.01), and *p-value (0.01–0.05). Hosmer–Lemeshow test = 0.72. Bold values represent the significantly associated variables.

## Discussion

Social phobia harms educational achievement by increasing school absentees and preventing students to participate in class, and this leads to a significant impairment of the emotional, psychological, social, and physical wellbeing of students. In this study, the prevalence of social phobia and its possible association with different factors were assessed. The result revealed that a remarkable proportion of students had a social phobia.

The finding of the current study showed that the prevalence of social phobia among high school adolescents in Northwest Ethiopia was 40.2% with (95%, CI: 37.0, 43.4%), which was consistent with the findings of other studies done in two areas of India; Sonitpur Assam and Karnataka, India, reported to be 38.3 and 39.7%, respectively, (33, 34). The reason for the agreement could be the similar screening tool used in both the previous and the current study called Social Phobia Inventory (SPIN) and the other reason could be the similar type of population; both the current and the Karnataka district were conducted in a rural type of populations (35, 36).

However, the prevalence of social phobia in this study was higher than in previous research findings done in Northeastern Ethiopia, high school students (27.5%) (10). The variation could be the difference in several female participants between the current and previous studies. In the previous study, only 39.6% and, in the current study, more than half (55.4%) were female students. Therefore, traits like submissive behaviors, avoidant personality, and shyness are more likely to be common in female students than males, the latter leading to the development of phobic symptoms (20). The current social phobia prevalence was also higher in studies done outside Ethiopia among high school adolescents in a comparative study between Arabian countries; Suhag Egypt, Abu Dhabi (UAE), and Abha (Saudi Arabia) were 13, 7.8, and 9.8%, respectively, (37). Similarly, the current study on social phobia prevalence is also higher than a study done in Abha Saudi Arabia in 2013 (11.7%), Ahmedabad India (12.8), Puducherry India (22.9), Swedish (10.6), Iran (6.2%), and Erbil, Kurdistan Region, Iraq high school students (31.25%) (15, 17, 20, 24, 38, 39). The possible reason for the variation may be due to differences in sociocultural, socioeconomic, measurement tools, type of study populations, and availability of health facilities between those countries and Ethiopia. In Ethiopia, the perceptions of adolescents toward shyness as a measure of politeness are a predominant cultural norm, skills of social interaction might not be well developed, and later adolescents could be easily distressed in social gatherings (10). People living in low socioeconomic countries like Ethiopia could have poor healthcare infrastructure and a shortage of trained health staff that delivers inadequate healthcare services; in turn, social phobia might not be early identified and treated (40). The tool assessments used in Abha Saudi Arabia, Sweden, and Iran were different from the tool used in the current study. Leibowitz Social Anxiety Scale test (LSAS), Social Phobia Screening Questionnaire (SPSQ), and DSM four diagnostic tool was used in Saudi Arabia and Sweden, respectively, while the current study was using Social Phobia Inventory (SPIN), which is a non-diagnostic self-administered screening tool, and this might overestimate the prevalence of social phobia among adolescents (41, 42).

On the contrary, the current study finding is lower than the previous study done among Abha Saudi Arabia in 2020 (45%) and Scotland UK high school students (53.8%) (16, 43). The discrepancy could be the difference in age of participants between the current study and Abha Saudi Arabia and the UK. In the current study, the mean age of participants was 18.49 ± 1.706 and only 54.6% of the students were less than 18 years old, whereas all of the participants in the UK and 93.3% in Saudi Arabia were less than 18 years old, in which social phobia is more likely common as youngers could have lack of social skills, attention, and learning problems (16, 17).

Regarding factors affecting social phobia, the female sex was significantly associated with higher rates of a social phobia than males. SP was nearly one and a half times more prevalent among females than males. These findings, supported by other studies in Ethiopia, Puducherry India, Sweden, and Iran, also reported that SP was more frequent in females than in males (10, 15, 20, 38). The reason could be females are not equally participated in all activities, and especially in Ethiopia their activity is limited at home only because of cultural influence when compared to males; in our culture, males dominated and received special care from their parents and as a result, females have felt uncomfortable in social gatherings (10, 44); in all developing countries, the perception of the community toward shyness and politeness as a measure of predominant cultural norm might have influenced the higher prevalence of social phobia among female students (20).

The present study also showed that social phobia was significantly associated with the presence of known chronic medical illnesses in high school adolescents. The odds of having social phobia were two times more common among students having a history of known chronic medical illness as compared with encounter parts. Similar findings were reported in Southeastern Ethiopia and Abha Saudi Arabia (14, 16). There are several possible reasons why students with known chronic medical illnesses may be experienced high levels of social phobia. First, parents of students with known chronic medical illnesses may show overprotective behavior that may risk the development of phobic symptoms (45, 46). Second, students with chronic medical illnesses may become socially anxious, because of an increased risk of being rejected by peers (47). Third, students with known chronic medical illnesses are also faced with dangerous stimuli, such as threatening symptoms of illness, for example, in case seizures may cause phobic symptoms (48).

Adolescents with a positive family history of mental illness had about 1.7 times more odds to have social phobia as compared with students who had no family history of mental illness. This finding is consistent with other findings done in a comparative study conducted between Egypt, Saudi Arabia, and the United Arab Emirates high school students and also among adolescent populations in South India (37, 49). This may be due to genetic factors and the influence of similar cultural and social practices. As highly anxious families have less social interaction with others, the adolescents’ exposure to various social gatherings might also be limited. In turn, this might have negatively affected the development of their social skills and thus made them susceptible to social phobia. In the process, students could not have learned that social situations are harmless (49).

When compared to low risky alcoholic drinkers, the odds of social phobia were two times more common among highly risky alcoholic drinkers. This was supported by a previous study conducted in Woldia Ethiopia and Nigeria (10, 26). The possible reason could be that highly risky current alcoholic drinkers may use alcohol frequently to self-medicate to relieve their fear, anxious feelings, and concerns of negative evaluation by others (26, 50).

Finally, students with poor social support had more than two times more likely to have SP as compared with students who had strong social support. This finding was supported by the previous study done in Ethiopia and Iran (10, 51). Social connectedness is useful for the development of self-confidence and good social skills. Therefore, if a student loses these skills later, they could be faced difficulties to cope with the situation when exposed to social gatherings (10, 52).

### Limitations of the study

There might be social desirability bias due to sensitive questions related to substance use. The other limitation is that as a cross-sectional study design was used, the current study design cannot show the direction of the association.

## Conclusion

In this study, the overall prevalence of social phobia was high. The distribution of SP among high school adolescents showed that it was higher in the female gender, students who have a history of y known chronic medical illness and family history of y mental illness, highly risky alcoholic drinkers, and poor social support. Therefore, early detection and adequate introversion are crucial to reducing the overall burden of social phobia among high school students. Extending mental health services and strengthening the existing counseling services in all high schools are recommended. The authors also recommended conducting longitudinal research to identify the cause-and-effect relationship of SP with different factors.

## Data availability statement

The raw data supporting the conclusions of this article will be made available by the authors, without undue reservation.

## Ethics statement

The studies involving human participants were reviewed and approved by University of Gondar Institutional Review Bored. Written informed consent to participate in this study was provided by the participants’ legal guardian/next of kin.

## Author contributions

GN conceptualized the study and involved in design, analysis, interpretation, report, and manuscript writing. MM, GD, and TZ made substantial contribution to conception, analysis, and interpretation of data, drafting the manuscript, and critical revision for important intellectual content. All the authors read and approved the final manuscript.

## Abbreviations

AOR: adjusted odds ratio
ASSIST: Alcohol, Smoking, and Substance Involvement Screening Test
CI: confidence interval
COR: crude odds ratio
DSM: Diagnostic and Statically Manual
EB: Ethiopian Birr
GC: Gorgonian Calendar
km: kilometer
OR: odds ratio
SP: social phobia
SPIN: Social Phobia Inventory
UAE: United Arab Emirates
UK: United Kingdom
USA: the United States of America
WHO: World Health Organization.
